# *Rickettsia felis* Infections, New Zealand

**DOI:** 10.3201/eid1801.110996

**Published:** 2012-01

**Authors:** Mei Yin Lim, Helen Brady, Tammy Hambling, Kerry Sexton, Daniel Tompkins, David Slaney

**Affiliations:** Institute of Environmental Science and Research Limited, Porirua, New Zealand (M.Y. Lim, H. Brady, T. Hambling, K. Sexton, D. Slaney);; Landcare Research, Dunedin, New Zealand (D. Tompkins)

**Keywords:** Rickettsia typhi, murine typhus, Rickettsia felis, indirect immunofluorescence assay, Western blotting, rickettsia, New Zealand

To the Editor: Members of the genus *Rickettsia* have garnered much attention worldwide in recent years with the emergence of newly recognized rickettsioses. In New Zealand, only *Rickettsia typhi* and *R. felis,* belonging to the typhus and spotted fever groups, respectively, have so far been found (*1*). *R. typhi*, primarily transmitted by the oriental rat flea (*Xenopsylla cheopis*), has a worldwide distribution and causes murine typhus in humans (*2*). At the end of 2009, a total of 47 cases of murine typhus had been recorded in New Zealand. In contrast, although the cat flea (*Ctenocephalides felis*) can carry *R. felis* in New Zealand (*3*), no human infections have been reported. However, because *R. felis* shares a similar clinical profile to murine typhus, infection can be mistaken for a suspected case of *R. typhi* (*4*).

Clinical suspicion of rickettsial infection is widely confirmed by serologic tests with the indirect immunofluorescence assay (IFA) being the standard test. However, antibodies against *R. felis* in human sera are known to cross-react with *R. typhi* in IFA (*5*). Western blot (WB) and cross-adsorption assays, in combination with IFA, can differentiate between several rickettsioses (*5**,**6*). We report on the trial in New Zealand of WB and cross-adsorption assays for differentiating retrospectively between past *R. typhi* and *R. felis* infections and evidence of *R. felis* infection in persons living in the country.

Serum samples were obtained from 24 volunteers from the Institute of Environmental Science and Research Limited, Porirua, New Zealand. Samples were tested using *R. typhi* IFA slides (Australian Rickettsial Reference Laboratory [ARRL], Geelong, Victoria, Australia). After incubation (37°C for 30 min), slides were washed 3 times, incubated with fluorescein-conjugated antihuman IgG, IgM, and IgA (ARRL), and washed again before examination. All samples were then tested by using an IgG IFA kit (Focus Diagnostics, Cypress, CA, USA) against typhus group (TG) *R. typhi* and spotted fever group (SFG) *R. rickettsii*.

TG-positive and SFG-negative serum samples may represent *R. typhi* infections, and SFG-positive and TG-negative serum samples may represent *R. felis* infections. Because *R. typhi* can cross-react with SFG rickettsiae (*7*), and *R. felis* with *R. typhi* (*5*), results that are TG positive and SFG positive may be caused by either rickettsiae. Positive reactivity may also represent overseas-acquired rickettsioses. Thus, WB and cross-adsorption assays using *R. typhi* (Wilmington) and *R. felis* (URRWXCal2) antigens (Unité des Rickettsies, Marseilles, France) were used to confirm any *R. typhi* or *R. felis* infections (*6*).

Antigens (2 mg/mL) were solubilized (100°C for 10 min) in 2× Laemmli buffer (*6*) and subjected to electrophoresis (20 μg/well; 20 mA, 2.5 h) through polyacrylamide gels (12.5% resolving; 4% stacking) (BioRad, Hercules, CA, USA). Resolved antigens were electroblotted (100 V for 1 h) onto 0.45-μm polyvinylidene difluoride membranes, which were blocked by using Tris-buffered saline with 0.1% Tween 20. Each antigen lane was divided into 2 strips before incubation (room temperature for 1 h) with serum (diluted 1:200). After three 10-min washes with 5% milk–Tris-buffered saline with 0.1% Tween 20, strips were incubated (room temperature, 1 h) with horseradish peroxidase–conjugated antihuman IgG (1:150,000; SouthernBiotech, Birmingham, AL, USA) and washed again. Enhanced chemiluminescent detection of bound horseradish peroxidase (ECL Plus; GE Healthcare, Buckinghamshire, UK) enabled identification of reactive band sizes with Precision Plus standards (BioRad). Cross-adsorption was carried out by incubating serum diluted 1:30 in boiled antigen (37°C for 5.5 h, then 4°C overnight) before centrifugation (10,000 × *g* for 10 min) (*6*). Supernatants were applied to WB strips and results compared with *R. typhi* and *R. felis* antisera.

Of the 24 serum samples, 3 (12.5%) were positive on the ARRL slides, and 11 (45.8%) showed IgG reactivity on Focus slides (Table). Of these 11 serum samples, 8 (33.3%) were SFG positive and TG negative, and 3 (12.5%) were SFG and TG positive. Of the 12 serum samples that showed some IFA reactivity, after cross-adsorption, none had specific reactivity against *R. typhi*, and 2 were confirmed as *R. felis*. Both volunteers recorded risk factors associated with *R. felis* infection. Six serum samples were indeterminate. Detectable antibodies remained after both cross-adsorptions, which may be caused by infection by *R. felis* and *R. typhi*, or other rickettsiae or cross-reactive pathogens (*5**,**8*).

**Table:** Serologic data and risk factors of volunteers that showed positive reactivity in rickettsial IFA, New Zealand*

Volunteer no.	IFA serologic titers				WB and cross-adsorption results	Risk factors in the past 4 years	
	ARRL kit†		Focus kit‡			Flea or animal contact	Traveled overseas
	*Rickettsia typhi*		*R. typhi*	*R. rickettsii*			
4	Neg		64	64	Indeterminate§	Rat, cat, dog	Yes
5	Neg		Neg	64	Indeterminate	Cat, dog	Yes
8	Neg		Neg	128	Neg¶	Flea, rat, cat, dog	Yes
9	Neg		Neg	64	Indeterminate	Flea, cat, dog	Yes
16	Neg		Neg	128	Indeterminate	Rat, cat, dog	Yes
17	Neg		Neg	128	*R. felis*	Rat, cat, dog	Yes
18	128		64	64	Neg	Cat, cow, hen	Yes
19	128		Neg	64	Indeterminate	Cat, dog	Yes
21	Neg		64	256	*R. felis*	Flea, cat, dog, possum	Yes
22	Neg		Neg	64	Neg	Cat, dog	Yes
23	256		Neg	Neg	Neg	Rat, cat, dog	No
25	Neg		Neg	128	Indeterminate	None	Yes

*IFA, indirect immunofluorescence assay; ARRL, Australian Rickettsial Reference Laboratory; WB, Western blot; neg, negative. †The cutoff titer for seropositivity was 128 as recommended by the manufacturer. ‡According to kit instructions, endpoint titers ≥64 and <256 indicate either past infection or early response to a recent infection, and ≥256 are considered presumptive evidence of recent or current infection. §Serum samples that still reacted with *R. typhi* and *R. felis* after cross-adsorption were classified as indeterminate responses. ¶Serum samples that showed no specific reactions to *R. typhi* and *R. felis* in the WB assay were classified as negative for *R. typhi* and *R. felis..*

Although IgG titers decline over time, detectable levels can remain for 4 years and thus exposure to *R. felis* may have occurred any time during this period (*9*). Because both *R. felis*–infected persons had traveled overseas within the past 4 years and *R. felis* has a wide distribution (*4*), overseas exposure is possible. *R. felis* is known to be prevalent in *C. felis* fleas, including in New Zealand (*3**,**4*). This prevalence and the high rate of cat and dog ownership have public health implications and support the recognition of *R. felis* as an emerging global health threat (*4*). Infection from *R. felis* in addition to *R. typhi* should be considered in the differential diagnosis of fever, headache, myalgia, and rash.
